# The Natural Course of Intermittent Exotropia over a 3-year Period and the Factors Predicting the Control Deterioration

**DOI:** 10.1038/srep27113

**Published:** 2016-06-03

**Authors:** Jeremy J. S. W. Kwok, Gabriela S. L. Chong, Simon T. C. Ko, Jason C.S. Yam

**Affiliations:** 1 Department of Ophthalmology, Tung Wah Eastern Hospital, Hong Kong; 2 Department of Ophthalmology and Visual Sciences, The Chinese University of Hong Kong, Hong Kong

## Abstract

The natural course of intermittent exotropia and the factors affecting its control has been unclear. We aim to report the natural course of our cohort of 117 Chinese children with intermittent exotropia and to identify baseline parameters that may have predictive value in the control deterioration of the disease. The visual acuity, spherical equivalent, compliance to orthoptic exercise, angle of deviation fusional convergence parameters and Newcastle Control Score were recorded for all children at baseline and at 3 years apart. Patients were divided into two groups according to the change in control over the 3 years: group 1 included patients who had no deterioration or had improvement in disease control; and group 2 were those who had deteriorated control or had undergone surgery. There were 77 patients (66%) in group 1 and 40 (34%) patients in group 2. Comparing the baseline parameters of the two groups, group 1 had statistically significantly smaller angle of deviation, larger fusional reserve, larger fusional recovery, and higher fusional reserve ratio (p < 0.05). Other baseline parameters were similar between the two groups. The baseline fusional parameters may have predictive value in determining the control of intermittent exotropia.

Intermittent exotropia (IXT) is a common entity among Chinese children^1^. Despite its high prevalence in daily practice, the natural history of IXT is still unclear. This may be because previous studies have a limited sample size or are retrospective in nature^2^,^3^,^4^.

Older studies seem to have no standardized method in assessing the control of IXT. Some considered the angle of deviation during a manifested exotropia to be less significant compared to the frequency with which it is manifested^5^. Others defined the control as the observed frequency with which the exo-deviation is manifested and along with the speed and ability to regain realignment after dissociation^6^. More recently, the assessment of the control has become more and more objective and standardized with the emergence of different point based systems^7^,^8^.

Other parameters apart from these conventional ones may be helpful in understanding the natural history of IXT. Recent studies demonstrated that fusional convergence parameters are associated with the control of IXT^9^,^10^. Conducting a prospective study with the Newcastle Control Score would be helpful to understand the factors predicting the natural development of IXT.

The purpose of this study is to determine the natural course of IXT and to identify fusional convergence parameters that may have predictive value in the change in the Newcastle Control Score in a 3-year period.

## Methods

This study protocol was carried out in accordance with the principles of the Declaration of Helsinki and was approved by Tung Wah Eastern Hospital Institutional Review Board. Written informed consent was obtained from all patients and their guardians.

### Subjects

All subjects in this study were Chinese patients under 18 years of age who were diagnosed with intermittent exotropia at Hong Kong Island Eastern cluster hospitals (Tung wah Eastern Hospital and Pamela Youde Nethersole Eastern Hospital) between June 2011 to February 2012. The second follow up was between 2014–2015 at 3-year period follow up.

Patients with any of the following criteria were excluded: non-Chinese ethnicity; a history of amblyopia (best-corrected visual acuity of <6/12 in one or both eyes or an interocular difference of at least 2 lines in the Snellen visual acuity chart); the presence of an A or V pattern, a small-angle intermittent exotropia (distance angle <10Δ); a convergence insufficiency-type intermittent exotropia (near angle more than 10Δ greater than distance angle); patients with a history of ocular surgical correction; and patients with developmental delay or neurologic problems.

### Assessment of control

Control was assessed by a single observer (GSLC). The NCS was documented and collected at presentation, preoperative assessment and at the 3-year follow up. For assessment, patients were divided into 3 control levels by the NCS. A score of 1–3 was graded as good control; 4–6 was graded as moderate control; and 7–9 was graded as poor control.

### Measurement of Angle of deviation and convergence parameters

Assessment of the angle of deviation and fusional convergence parameters were done by a single observer (GSLC). The angle of deviation was measured using a prism bar cover test for distance (6 m) and for near (1/3 m). The patients also underwent full ophthalmologic examination, including measurement of near stereopsis using the Frisby test. For patients with refractive errors not requiring correction with spectacles, best-corrected visual acuity was achieved with trial frames for the examination.

The documentation of the angle of deviation for near and angle of deviation for distance was measured before the measuring of fusional convergence parameters. Fusional convergence parameters including the fusion breakpoint or fusional reserve for near and distance was recorded. Fusional recovery point at near and distance was also measured. The total fusional convergence amplitude at near was calculated by adding the fusional reserve at near with the angle of deviation at near. The total fusional convergence amplitude for distance was calculated by adding the fusional reserve at distance with the angle of deviation at distance.

As for the fusional reserve ratio, the fusional reserve ratio for near was calculated by dividing the fusional reserve at near with the angle of deviation at near. The fusional reserve ratio at distance was calculated by dividing the fusional reserve at distance with the angle of deviation at distance. For example, the fusional reserve ratio in a patient having a convergence reserve of 40Δ and an angle of deviation of 20Δ would be 40Δ/20Δ which results in 2.

The definition of the fusional parameters and method of measurement was reported by Yam *et al.*^10^. The angle of deviation was not neutralized before the measurement. The observer was to determine whether the patient could maintain the alignment in free space before the fusional reserve was measured. A based out prism bar was applied and power was gradually increased until the patient could no longer maintain fusion (diplopia or observed exodeviation). This prism power at the break point was the amplitude of fusional convergence or the fusional reserve. The prism bar was then decreased in power until fusion was re-established. This was designated the point of fusional recovery. For the purpose of our analysis, patients whose fusion was not broken at 40Δ was given a fusional reserve of 45Δ and the fusional recovery was not measured. If the patient was unable to recover in free space, the recovery point was to be recorded as 0.

### Data Analysis

Patients were divided into 2 groups for assessment for risk factors. Group 1 were patients who were static or improved in their control level. Groups 2 were patients who have deteriorated in control level or patients who have undergone surgery between the 2 assessments. Age of presentation, gender, visual acuity, refractive errors, fusional convergence parameters, and changes in the size of exodeviation for near and distance were compared between the 2 groups. Data that was normally distributed were compared with unpaired independent T-test. Mann Whitney-U test will be used to compare data that does not follow a normal distribution. All analysis and calculations will be done with IBM SPSS (StatLab, version 22; SPSS Inc, Chicago, IL).

## Results

### Combined ungrouped statistics

There were 117 patients who met the inclusion criteria. The mean time of follow up was 26.7 +/− 8.3 months. The mean age of presentation was 8.6 (+/−3.6) years old, there were 73 (62.4%) girls and 44 (37.6%) boys. The mean visual acuity by logMAR was 0.016 (+/−0.08) in the left eye and 0.020 (+/−0.08) in the right eye. The mean spherical equivalent in the right eye was −1.28 (+/−2.34) diopters and −1.36 (+/−2.50) diopters in the left eye.

The overall mean NCS score was 4.55 (+/−1.64) at presentation. The patients were divided into 3 control levels for assessment. At baseline, there were 25 patient with good control (21.4%), 76 with moderate control (65.0%), and 16 with poor control (13.7%). During the 3-year visit, 30 (25.6%) had good control, 51 (43.6%) had moderate control, 14 (12.0%) had poor control, and 22 (18.8%) had undergone surgery.

The angle of deviation and fusional convergence parameters at presentation and at 3-year follow up are summarized in Table 1.

### Surgical patients

There were 22 patients who underwent surgery during the 3-year follow up period. The mean age of diagnosis with IXT in the surgical patients was 7.59 +/− 3.0 years old. There were 7 (31.8%) males and 15 (68.2%) females. The average NCS was 6.0 +/− 1.5 at initial assessment and 6.6 +/− 1.1 at pre-operative assessment. Control level based on the NCS at initial assessment showed that 1 (4.5%) patient had good control, 11 (50.0%) had moderate control and 10 (45.5%) had poor control. The average angle of deviation at near was 22.68 +/− 6.4Δ at initial assessment and 24.45 +/− 5.9Δ at pre-operative assessment. The average angle of deviation for distance was 26.27 +/− 6.1Δ at initial assessment and 26.81 +/− 5.9Δ at preoperative assessment.

The NCS was static in 45.5% of the cases with 36.2% deteriorating in a NCS of 1. 13.6% of cases deteriorated in NCS by >2. The angle of deviation at near was static in 81.8% and 18.2% of the cases had a deterioration of >5Δ between the initial assessment and the pre-operative assessment. The angle of deviation for distance was static in 95.5% and 4.5% deteriorated >5Δ between the initial assessment and the pre-operative assessment.

### Non-surgical patients

The 95 non-surgical patients had a mean age of 8.8 years (+/−3.7) at initial assessment. There were 37 (38.9%) male and 58 (61.1%) females. The average NCS was 4.19 at baseline and 4.03 at follow-up. There were 27 (28.4%) patients graded with good control, 62 (65.3%) graded with moderate control, and 6 (6.3%) who were graded with poor control in the initial assessment. At third year assessment, 30 (31.6%) patients were graded with good control, 51 (53.7%) with moderate control, and 14 (14.7%) with poor control.

The average angle of deviation at near was 17.7Δ, angle of deviation for distance was 21.6Δ at initial assessment. At follow up, the average angle of deviation at near was 17.1Δ and angle of deviation at distance was 21.3Δ. For the angle of deviation at near, 22 (23.2%) patients had an increase of >5Δ, 53 (55.7%) patients remain within +/−5Δ of initial presenting angle, and 20 (21.0%) had an decrease of >5Δ at follow up. There were 21 (22.1%) patients with a >5Δ increase in angle of deviation for distance, 51 (53.7%) was static within +/−5Δ of the presenting angle, and 23 (24.2%) had a decrease in >5Δ between the 2 assessments. Including the surgical patients that are considered to be in the deteriorating group, most patients of our series were either static or have improved (angle of deviation at near 62.4%, angle of deviation at distance 63.2%) in control over the 3-year follow up period.

The average fusional convergence parameters at initial assessment were: fusional reserve at near: 17.6Δ, fusional reserve at distance: 7.3Δ; fusional recovery at near: 13.1Δ, fusional recovery at distance: 4.7Δ. The average fusional convergence parameters at 3-year follow up were: fusional reserve at near: 16.4Δ, fusional reserve at distance: 6.1Δ; fusional recovery at near: 12.3Δ, and fusional recovery at distance: 3.6Δ.

The fusional reserve at near was static within +/−5Δ in 44 (46.3%) patients, increased by >5Δ in 23 (24.2%) patients, and decreased >5Δ in 28 (29.4%) patients. The fusional reserve at distance was static +/−5Δ in 53 (55.7%) patients, increased >5Δ in 18 (18.9%) patients, and decreased >5Δ in 24 (25.4%) patients.

The fusional recovery at near was static within +/−5Δ in 48 (50.5%) patients, increased >5Δ in 24 (25.2%) patients, and decreased >5Δ in 23 (24.2%) patients. The fusional recovery at distance was static within +/−5Δ in 66 (69.5%) patients, increased >5Δ in 11 (11.6%) patients, and decreased >5Δ in 18 (18.9%) patients.

### Group 1 (Static or improved in control level) vs group 2 (deterioration or surgery) statistics

At the third year assessment, there were 77 patients (65.8%) in group 1 and 40 (34.2%) patients in group 2. Within group 2, 18 had deteriorating control (45.0%) and 22 patients received surgery (55.0%).

Demographics were analyzed by 2-tailed independent T-test. The average age of the patients in group 1 and group 2 was 9.1 +/− 3.8 years of age and 7.4 +/− 3.0 years of age respectively (p = 0.019). There were 30 males (39.0%) and 47 females (61.0%) in group 1; and 14 males (35.0%) and 26 females (64.0%) in group 2 (p = 0.68). There was a general myopic shift in all patients. The refractive error change and visual acuity is not statistically significant between the 2 groups and is summarized in Table 2.

The angle of deviation and fusional convergence parameters were compared with the Mann Whitney-U test. There were statistical significant differences between group 1 and group 2 in terms of the angle of deviation at near, angle of deviation at distance, fusional reserve for near, fusional reserve for distance, fusional recovery at near, and fusional recovery at distance. The fusional reserve ratio for near and distance was also significantly different between the 2 groups. However, there was no statistical significant difference between the total convergence amplitude for near or distance. The summary is in Table 3.

### Orthoptic Exercise vs control

A Chi-square test was performed for the association between orthoptic exercise and control of IXT. There was no statistical significant difference between the groups (p = 1.0).

## Discussion

As there is limited understanding of the risk factors and longitudinal progression of IXT, the purpose of this paper was to determine risk factors that are associated with the deterioration in the control of IXT and to look at the changes in the angle of deviation and fusional convergence parameters in non-surgical cases. We found that the size of the fusional reserve parameters, the size of the angle of deviation, and the control based on the NCS was relatively stable in IXT over a 3-year period.

### Changes in size of IXT and fusional convergence parameters

The findings of previous studies on the natural history and angle of deviation showed variable results. Hiles *et al.* reported a mean decrease in distance exodeviation of 5Δ in 48 unoperated patients with a mean FU of 11.7 years. The average change overall was improved by of 5Δ and that 81% of patients had no deterioration^3^. Romanchuk reported that in 109 patients followed up for 9 years, 19% of the patients improved in size of exodeviation by >10Δ, 58% remained the same and 23% worsened by 10Δ^11^. Noorden and Campos had the most deteriorated case series with 75% of 51 untreated patients demonstrating an increase in magnitude of deviation and/or progressive loss of binocular function for an average follow-up of 3.5 years^12^. In our series we report an average decrease in 0.59 +/− 9.5Δ in the angle of deviation at near and a decrease in 0.39 +/− 10.0Δ in the angle of deviation at distance over 3 years among nonsurgical patients. This was similar from average improvements in size of exotropia at distance of 1.1 +/− 10.6Δ at 1st year and 3.2 +/− 13.4Δ at 3 years in Chia *et al.*^2^. The decrease in the angle of deviation may be because stable patients were improving over time whereas the patients who were deteriorating would have undergone surgery.

Chia *et al.* reported that the angle of deviation at distance was within +/−5Δ of presentation in 48% of 89 non-surgical patients, 32% would have an improvement of >5Δ, and 20% deteriorated for >5Δ at 3 years of follow-up. The angle of deviation at near was static within +/−5Δ in 47% of the patients and deteriorated by >5Δ in 35%^2^ of the patients. Similarly, our non-surgical patients also showed that the angle of deviation at distance and angle of deviation at near would remain static in most patients (angle of deviation at distance within 5Δ in 54% of the patients; angle of deviation at near within 5Δ in 56% of the patients), and that 24.2% of the patients would have an improvement in angle of deviation at distance of >5Δ.

Recent reports have suggested that the fusion convergence had an impact on the control of IXT. Hatt *et al.* published data on correlation between fusional convergence and a 5-point scale system of control in a series with 64 children with IXT. The fusional reserve at distance was 7 +/− 8Δ, the fusional reserve at near was 21 +/− 14Δ, fusional recovery point at distance was 2 +/− 5Δ, and the fusional recovery point at near was 11 +/− 10Δ^9^. Yam *et al.* was also able to correlate the control by NCS with the fusional convergence parameters in Chinese children^10^. Both Hatt *et al.* and Yam *et al.* reported that there were significant correlations between fusional convergence parameters especially the fusional reserve ratio and the control. The assessment of the control and time for surgery is difficult for IXT. From our study we think that fusional convergence is a helpful tool in determining the trend in gradual loss of control of IXT and thus can aid in deciding the best timing for surgical intervention.

### Risk factors for change in control of IXT over 3 years

Independent sample T-test showed that patients in group 2 that deteriorated in control of IXT had a significantly younger age of presentation. They had a significantly lower fusional reserve at near, fusional reserve at distance, fusional recovery at near, fusional recovery at distance, fusional reserve ratio at distance, and fusional reserve ratio at near by Mann-whitney U test. There is no difference between the 2 groups in terms of gender, refractive error changes, total convergence amplitude at near, and total convergence amplitude at distance. Previously, Hatt *et al.* and Yam *et al.* had published cross section correlations between fusional reserves and clinical control by scoring systems. However our study is the first to associate fusional reserves with control over a 3-year study period^9^,^10^.

Theoretically, orthoptic exercise would strengthen convergence and is widely given to patients due to its non-invasiveness. Rutstein and Corliss reported findings in a 3Δ decrease in exodeviation in 73 patients with an average of 10 year follow up^13^. Others suggest patients with orthotropic exercise showed no worsening of angle of deviation over a mean of 5.5 years of follow up. Nasz published that 21% of the reported 138 cases had received some form of medical intervention but there was no significant difference in progression between patients who received medical therapy and those who did not^4^. Orthoptic exercise was taught to all our patients with IXT but only few had volunteered good compliance. There was no statistical significance between those who were compliant to orthroptic exercise and those who did not with Chi-square test.

### Limitations

One limitation in our study is that the collected data may be confounded by daily variability. A single NCS score has not been validated as representative of a patient’s overall control. Hatt *et al.* previous publish that stereopsis and control can even vary within a single day^14^,^15^. This study was also limited by the lack of normal controls for comparison. Nevertheless, we are following the cohort longitudinally to observe the trend. Comparison with the normal control was not essential. Finally, the NCS system has inherent limitations: defining “good” control as an NCS of 0–3 is arbitrary. The NCS was used to standardize the assessment but not completely eliminate subjectivity.

## Conclusion

Over a 3-year period, about one-third of Chinese children in our study with intermittent exotropia deteriorated in the control. The baseline fusional convergence parameters may act as predictive factors in determining the trend of the control.

## Additional Information

**How to cite this article**: Kwok, J. J. S. W. *et al.* The Natural Course of Intermittent Exotropia over a 3-year Period and the Factors Predicting the Control Deterioration. *Sci. Rep.* **6**, 27113; doi: 10.1038/srep27113 (2016).

## Figures and Tables

**Table 1:** Measurements of angle of deviation and fusional convergence parameters at initial assessment and at 3-year follow up for surgical and non-surgical cases.

	Initial assessment(n = 117)	3-year follow up: No surgery(n = 95)	3-year follow up: post-surgery(n = 22)
Angle of deviation at near (Δ)	18.6 (+/−8.1)	17.1 (+/−7.9)	9.5 (+/−8.5)
Angle of deviation at distance (Δ)	22.5 (+/−8.0)	21.3 (+/−8.8)	8.2 (+/−8.8)
Fusional reserve at near (Δ)	16.4 (+/−11.0)	16.4 (+/−9.7)	18.0 (+/−9.8)
Fusional reserve at distance (Δ)	6.7 (+/−7.1)	6.13 (+/−7.1)	11.2 (+/−7.6)
Fusional recovery at near (Δ)	12.0 (+/−9.4)	12.3 (+/−8.7)	13.8 (+/−7.3)
Fusional recovery at distance (Δ)	4.3 (+/−5.5)	3.6 (+/−5.4)	8.2 (+/−6.2)
Total convergence amplitude at near (Δ)	35 (+/−12.6)	33.5 (+/−9.5)	27.5 (+/−10.8)
Total convergence amplitude at distance (Δ)	29.2 (+/−9.1)	27.4 (+/−8.7)	19.4 (+/−11.9)
Fusional reserve ratio at near	1.43 (+/−2.97)	1.03 (+/−2.01)	2.9 (+/−3.7)
Fusional reserve ratio at distance	0.44 (+/−0.73)	0.20 (+/−2.93)	2.3 (+/−3.25)

**Table 2:** Comparison of visual acuity and refractive errors of Group 1 (deteriorated patients) vs Group 2 (patients with no deterioration).

	Group 1: No Deterioration in Control (n = 77)	Group 2: Deteriorated in Control (n = 40)	P−value
Right eye visual acuity (logMAR) at initial	0.014	0.033	0.257
Right eye spherical equivalent at initial (diopters)	−1.25	−1.32	0.886
Right eye spherical equivalent change over 3 years (diopters)	−0.63	−0.63	0.978
Left eye visual acuity (logMAR) at initial	0.012	0.024	0.462
Left eye spherical equivalent at initial (diopters)	−1.43	−1.23	0.694
Left eye spherical equivalent change over 3 years (diopters)	−0.57	−0.74	0.635

Comparisons were made by 2-tailed independent T-test.

**Table 3:** Comparisons of measurements of angle of deviation and fusional convergence parameters between group 1 and group 2 patients with Mann-Whitney U test.

	Group 1: No Deterioration in Control (n = 77)		Group 2: Deteriorated in Control (n = 40)		U value	P-value (two-tailed)
	**Mean**	**Median**	**Mean**	**Median**		
Angle of deviation at near (Δ)	17.8	16	20.2	20	1144.5	0.022
Angle of deviation at distance (Δ)	21.5	20	24.4	25	1142.0	0.020
Fusional reserve at near (Δ)	18.5	18	12.4	10	1055.0	0.005
Fusional reserve at distance (Δ)	8.0	6	4.2	2	1047.0	0.004
Fusional recovery at near (Δ)	13.7	14	8.8	6.5	1072.5	0.007
Fusional recovery at distance (Δ)	5.4	4	2.2	0	1024.0	0.002
Total convergence amplitude at near (Δ)	36.3	34	32.6	29.0	1259.0	0.106
Total convergence amplitude at distance (Δ)	29.6	29	38.6	26.0	1452.0	0.614
Fusional reserve ratio at near	1.66	0.32	0.99	0.09	981.0	0.001
Fusional reserve ratio at distance	0.56	1.0	0.20	0.58	926.0	0.000

## References

[b1] Lin S. *et al.* Alcohol use and positive screening results for depression and anxiety are highly prevalent among chinese children with strabismus. American journal of ophthalmology 157, 894–900, 10.1016/j.ajo.2014.01.012 (2014). 24445033

[b2] Chia A., Seenyen L. & Long Q. B. A retrospective review of 287 consecutive children in singapore presenting with intermittent exotropia. J AAPOS 9, 257–263, 10.1016/j.jaapos.2005.01.007 (2005). 15956946

[b3] Hiles D. A., Davies G. T. & Costenbader F. D. Long-term observations on unoperated intermittent exotropia. Archives of Ophthalmology 80, 436–442, 10.1001/archopht.1968.00980050438006 (1968). 5674800

[b4] Nusz K. J., Mohney B. G. & Diehl N. N. The course of intermittent exotropia in a population-based cohort. Ophthalmology 113, 1154–1158, 10.1016/j.ophtha.2006.01.033 (2006). 16647128

[b5] Rosenbaum A. & Stathacopoulos R. Subjective and Objective Criteria for Recommending Surgery in Intermittent Exotropia. American Orthoptic Journal 42, 46–51 (1992).

[b6] Rosenbaum A. & Santiago A. P. In Clinical Strabismus Management 163–175 (WB Saunders Company, 1999).

[b7] Buck D. *et al.* The Use of the Newcastle Control Score in the management of Intermittent Exotropia. British journal of ophthalmology 91, 215–218, 10.1136/bjo.2006.097790 (2007). 17020901 PMC1857598

[b8] Haggerty H., Richardson S., Hrisos S., Strong N. P. & Clarke M. P. The Newcastle Control Score: a new method of grading the severity of intermittent distance exotropia. The British Journal of Ophthalmology 88, 233–235, 10.1136/bjo.2003.027615 (2004). 14736781 PMC1772020

[b9] Hatt S. R., Leske D. A., Mohney B. G., Brodsky M. C. & Holmes J. M. Fusional convergence in childhood intermittent exotropia. American journal of ophthalmology 152, 314–319, 10.1016/j.ajo.2011.01.042 (2011). 21621744 PMC3170040

[b10] Yam J. C. *et al.* A prospective study of fusional convergence parameters in Chinese patients with intermittent exotropia. J aapos 17, 347–351, 10.1016/j.jaapos.2013.03.023 (2013). 23911127

[b11] Romanchuk K. G., Dotchin S. A. & Zurevinsky J. The natural history of surgically untreated intermittent exotropia-looking into the distant future. J aapos 10, 225–231, 10.1016/j.jaapos.2006.02.006 (2006). 16814175

[b12] Noorden G. K. V. & Campos E. C. In Binocular Vision and Ocular Motility Ch. 17, 359 (Mosby, 2002).

[b13] Rutstein R. P. & Corliss D. A. The clinical course of intermittent exotropia. Optometry and vision science : official publication of the American Academy of Optometry 80, 644–649 (2003). 14502045 10.1097/00006324-200309000-00009

[b14] Hatt S. R., Mohney B. G., Leske D. A. & Holmes J. M. Variability of stereoacuity in intermittent Exotrpia. American journal of ophthalmology 145, 556–561, 10.1016/j.ajo.2007.10.028 (2008). 18201680 PMC2276647

[b15] Hatt S. R., Mohney B. G., Leske D. A. & Holmes J. M. Variability of control in intermittent exotropia. Ophthalmology 115, 371–376.e372, 10.1016/j.ophtha.2007.03.084 (2008). 17629562 PMC2774356

